# A systematic literature review of the role of trust and security on Fintech adoption in banking

**DOI:** 10.1016/j.heliyon.2023.e22980

**Published:** 2023-11-29

**Authors:** Johan Ariff Jafri, Syajarul Imna Mohd Amin, Aisyah Abdul Rahman, Shifa Mohd Nor

**Affiliations:** a Faculty of Economics and Management, Universiti Kebangsaan Malaysia, 43600, Selangor, Malaysia; b Institute of Islam Hadhari, Universiti Kebangsaan Malaysia, 43600, Selangor, Malaysia

**Keywords:** Fintech, Systematic literature review, Consumer, Trust, Security, TCCM framework

## Abstract

Fintech's development has amplified cybercrime, prompting trust and security concerns in banking. While earlier research predominantly viewed Fintech adoption through a tech-centric lens, emphasising its benefits, there is a paucity of studies on cognitive resistance arising from Fintech controversies. This review synthesises previous Fintech literature on behavioural intentions in banking, emphasising the role of trust, security, and other factors, and highlights existing research gaps. Utilising the ROSES (RepOrting standards for Systematic Evidence Syntheses) framework, a Systematic Literature Review was conducted, analysing 26 articles from Scopus and Web of Science (WoS) databases (2009–2022). Thematic analysis produces five primary themes (UTAUT2 variables; risk; trust; quality; and other), branching into 24 sub-themes. The weight analysis emphasises the best well-utilised predictors like performance expectancy, trust, security, perceived usefulness, and attitude. In addition, the review identifies research gaps and offers recommendations for future studies using the TCCM (Theory, Context, Constructs, and Method) framework. This research provides insights to Fintech companies and regulatory authorities on the preferred attributes of Fintech services that can enhance their adoption within the banking sector.

## 1 Introduction

Financial technology, or Fintech, integrates advanced digital technologies such as blockchain, data analytics, and sophisticated advisory technology [1]. Fintech lenders use big datasets and artificial intelligence (AI) or machine learning (ML) algorithms to speed up credit decisions, offering near-instant results [2]. Fintech firms outperform traditional financial institutions in efficiency and user experience [3]. The evolution of Fintech comprises four stages [4,5]. Fintech 1.0 (1866–1967) featured analogue financial services. Fintech 2.0 (1968–2008) saw the digitisation of the sector, driven by advances in transactional and communicative digital technologies. Fintech 3.0 began in 2009, introducing a surge in digital services, startups, cryptocurrencies, and smartphone usage in advanced economies. Fintech 3.5 sees emerging markets adopt these technologies, advancing financial inclusion and growth. Starting in 2018, Fintech 4.0 emerged with the introduction of non-fungible tokens (NFTs) and the rise of Neobanks.

Fintech development is transforming the banking landscape from standard online banking to feature-rich mobile apps, such as fund transfers, bill payments, cross-border remittance, robo-advisory, and wealth management [6]. Bank branches have declined from nearly 100,000 in 2009 to 79,974 by 2020 [7], reflecting the shift to digital. Fintech adoption in 27 countries has also surged, from 16 % in 2015 to 64 % in 2019 [8]. Despite technological advancement, Fintech exposes users to multifaceted risks, including ambiguous legal frameworks, regulatory gaps, compromised data privacy, and rapidly growing financial fraud. However, Fintech innovations expose users to risks such as ambiguous legal frameworks, privacy breaches, and financial fraud. In 2013, a cyber-attack by the Carbanak gang on more than 100 banks caused a loss of $1 billion [9]. By 2021, Cybersecurity Ventures anticipated a ransomware attack would occur every 11 s, up from every 14 s in 2019 to 40 s in 2016 [10]. Some digital banks have failed in the U.S. and Japan, while others have suffered losses from significant data breaches, hindering widespread Fintech adoption [11]. The divergence between Fintech's benefits and threats prompted research into consumer perceptions and intention to adopt Fintech services in the banking sector [12,13,14].

Despite the growing concern about Fintech, its long-term viability remains under examination [15]. Most Fintech adoption studies have centered on the benefits of technology, utilising frameworks such as the Technology Acceptance Model (TAM), Diffusion of Innovation, and Unified Theory of Acceptance and Use of Technology (UTAUT). There needs to be more research into cognitive resistance driven by external factors, such as during intensified cybersecurity incidents [16,17]. The escalation of cyberattacks and the vulnerabilities of information security systems jeopardise consumer trust and perceived security, leading to widespread hesitation in adopting Fintech services [18,19,20]. It is clear that while technological features are crucial, integrating technology adoption theories with trust and security elements is imperative, shedding light on Fintech's advantages and disruptive consequences [21].

Prior studies have examined the role of trust and security in various aspects of Fintech adoption, including blockchain technology [22,23] and robo-advisors [24]. In the context of banking, studies have explored trust and security implications in diverse settings, such as cloud computing in Malaysia using the integrated theory of TAM-DTM [25], smartphone banking in South Korea employing the TAM [26], mobile banking in India by integrating the TAM-SRT-UTAUT [27], and mobile banking in Egypt by considering TAM and the Service-Dominant (S-D) logic. Additionally, a study on digital-only banks in Malaysia incorporated the Mental Accounting Theory (MAT), Network Externalities Theory (NE), and Commitment-Trust Theory (CTT) [11]. However, this research is fragmented, scattered across different contexts and theories, needing a unified analytical framework to fully comprehend the impact of trust and security on Fintech behavioural intention in the banking sector. To gain a more comprehensive understanding of how trust and security factors influence Fintech behavioural intention in banking, a systematic literature review is imperative.

A systematic literature review (SLR) examines a formulated question using systematic and explicit methods to identify, select and critically appraise relevant research [28]. SLR allows the discovery of patterns of prior results while comprehending the depth and details of the existing knowledge, thus identifying gaps needed [29]. Moreover, SLR is a unifying tool, consolidating diverse theories, contextual nuances, and empirical discoveries from various studies. The current study employs the SLR technique, guided by the central research question: What is the role of trust and security in influencing Fintech behavioural intention in banking? The review aims to address theoretical gaps in the fifth fold. First, it identifies predominant and underutilised theories related to Fintech behavioural intention. Second, it emphasises the importance of integrating multidisciplinary theories to incorporate the role of trust and security in this context. Third, it distinguishes the various dimensions of trust, each contributing to understanding Fintech adoption. Fourth, it unveils that security constitutes a perceived risk dimension concerning banking customers. Finally, it ascertains the dynamic impact of trust and security, considering both direct and indirect (moderating and mediating) effects on Fintech's behavioural intention.

Previous SLRs on Fintech in banking have focused on Fintech concepts [30], digital payment [31], and Blockchain technology [32]. This study extends past SLR studies on the adoption of Fintech in banking by 1) synthesising the role of trust, security, and other determinants, 2) producing weight analysis, 3) providing thematic analysis, 4) identifying literature gaps, and 5) suggesting future research agenda based on the TCCM framework. The thematic analysis significantly advances existing research by elucidating commonalities among variables, which is crucial for refining and developing theoretical models, minimising variable redundancy, and enhancing the models' practical effectiveness. The findings will serve as a conduit, harmonising the theoretical framework with different countries’ unique characteristics and banking services. The weight analysis suggests well-utilised, promising, and experimental predictors that could be a reference for future studies in developing a Fintech adoption determinants framework. SLR and weight analyses provide valuable insights into the features and attributes that users prioritise in Fintech services, offering insights into user-accepted features crucial for the successful development and implementation of Fintech-based services. This comprehensive approach delves deeper into the current body of knowledge, ultimately identifying critical research gaps in Fintech adoption. Using the TCCM approach, this study suggests future research agendas based on theory, construct, context, and method [33].

The remaining sections of this review are organised as follows. The next section discusses the methodology used, followed by a discussion of findings and synthesis of selected literature. Next, it discusses potential future research directions, limitations, and the study's conclusion.

## 2 Methodology

### 2.1 Review protocol – ROSES

This article is guided by Reporting standards for Systematic Evidence Syntheses (ROSES). ROSES offer a better SLR technique for methodological-based articles such as social science [29]. It is designed for systematic review and maps to ensure that material is provided at the right level of detail, uses consistent terminology, and offers fundamental methodological direction [34]. It begins with formulating research questions, followed by systematic searching strategies (identification, screening, eligibility), appraisal of the quality of the selected articles, and data abstraction and analysis.

### 2.2 Formulation of research questions

The review formulated the research questions based on PICo (Population or Problem, Interest, and Context), as Shaffril et al. [35] suggested. Based on these concepts, three main aspects are included in the review (Table 1). These concepts will guide us in developing the main research question: What are the Fintech behavioural intention determinants? What is the role of trust and security in influencing Fintech intention?

**Table 1:** Aspects based on PICo concept.

Concept	Aspect
Population	Consumer.
Interest	Role of trust, security, and other determinants of intention to adopt Fintech-based innovations in banking.
Context	Global.

### 2.3 Systematic searching strategies

#### 2.3.1 Identification

Identification is searching any related terms, synonyms, and variations for the study's main keywords: trust, security, Fintech, and online banking. Based on these keywords, non-banking articles were excluded. This study enhances the primary keywords through references to sources such as encyclopedias, thesaurus, keywords from previous literature, terms recommended by well-established databases like Scopus, and insights from experts [35]. It aims to provide more options for selected databases searching for more related articles. The review relied on two primary journal databases – Scopus and Web of Science (WoS). Scopus is one of the most extensive abstracts and citation databases of peer-reviewed literature, with more than 25,100 journals [36]. It offers affiliations, the number of publications and their bibliographic data, and details on the number of citations. WoS enables the user to acquire, analyse, and disseminate database information. It includes 1.9 billion cited references from over 171 million records [37]. These databases were selected based on their perceived relevance and capacity to offer access to the most influential journals encompassing the knowledge map's subject matter. This choice was made to access the most impactful sources and content related to the research field. Furthermore, these two databases are frequently employed in bibliometric analyses and work together in a complementary manner [38]. Search strings on Scopus and Web of Science databases (WoS) were developed in December 2022 (Table 2) after all relevant keywords managed to be determined. Current research successfully retrieved 409 articles (185 Scopus; 224 WoS).

**Table 2:** Keywords and search strings.

Databases	Keywords and search strings
Scopus	TITLE-ABS-KEY ((“trust” OR “consumer* trust”) AND (“Adoption” OR “Acceptance” OR “Diffusion” OR “Usage” OR “Satisfaction” OR “Intention” OR “Use Behaviour” OR “Use Behaviour”) AND (“securit*" OR “data securit*" OR “data securit* threa*" OR “cybersecurit*" OR “information securit*" OR “cyberattack*") AND (“M-Banking” OR “Mobile Banking” OR ″E-Banking” OR “Electronic Banking” OR “Internet Banking” OR “Online Banking"))
Web of Science	TS = ((“trust” OR “consumer* trust”) AND (“Adoption” OR “Acceptance” OR “Diffusion” OR “Usage” OR “Satisfaction” OR “Intention” OR “Use Behaviour” OR “Use Behaviour”) AND (“securit*" OR “data securit*" OR “data securit* threa*" OR “cybersecurit*" OR “information securit*" OR “cyberattack*") AND (“M-Banking” OR “Mobile Banking” OR ″E-Banking” OR “Electronic Banking” OR “Internet Banking” OR “Online Banking"))

#### 2.3.2 Screening

Screening is the systematic searching strategy that includes or excludes articles from the review. This study screened 409 articles by choosing the criteria for article selection via the automatic sorting function available in the database. Only article journals with empirical data are selected (Table 3). Next, the search efforts focused only on English publications articles to make them easier to translate. This choice was made to facilitate translation and comprehension, as opting for studies in languages not understood by the researchers could lead to confusion, limited understanding, and the requirement for additional resources in terms of time and cost. This decision aimed to streamline the research process and ensure effective communication and comprehension of the selected literature [39]. Researchers should determine the range of periods they can review as it is almost impossible to review all the existing published articles [40]. Fourteen years, 2009–2022, an adequate period to see the evolution of research and related publications is selected. 2009 was selected because Fintech 3.0 only evolved after 2008 in developed and developing countries [41]. Traditional banks have begun to apply Fintech and digital applications due to the establishment of Internet financial services [42]. This process excluded 125 articles and removed 60 duplicate articles. The remaining 224 articles were used for the eligibility process.

**Table 3:** The inclusion and exclusion criteria.

Criteria	Inclusion	Exclusion
Literature type	Article journals (Empirical data)	Systematic review articles, review articles, meta-analyses articles, book series, chapter in the book, conference proceeding.
Language	English	Non-English
Timeline	>2008	<2009

#### 2.3.3 Eligibility

The 224 articles were manually monitored to ensure they aligned with the criteria by reading the title and abstract. 198 articles were excluded for several reasons, like focusing on other than behavioural intention to adopt Fintech in banking (perception) and conceptual (not empirical) studies. In addition, the review excluded unconvincing articles that did not examine the roles of trust and security in banking. Only 26 articles were selected: 14 (WoS) and 12 (Scopus).

#### 2.3.4 Quality appraisal

**Fig. 1:**
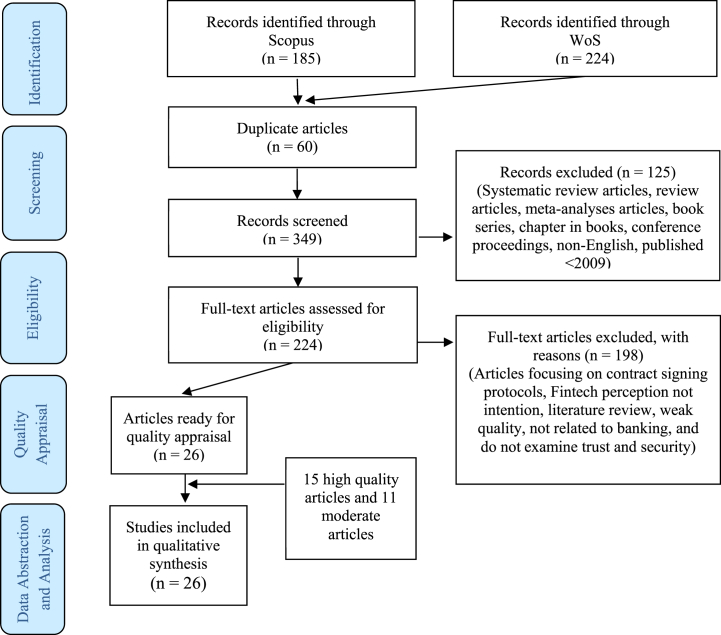
The flow diagram.

The selected 26 articles were presented to three experts in Fintech, banking, and consumer behaviour for quality assessment of the articles' content. The articles’ quality is categorised into high, moderate, and low. The experts use four commonly used criteria, namely credibility, dependability, confirmability, and transferability, to categorise the quality of qualitative research [43]. When an article meets all four criteria, it is deemed high quality; however, if only half of the criteria are met, it is only considered moderate quality. Only high and moderate articles should be reviewed, provided all authors must mutually agree. This process ranked 15 articles as high and 11 articles as moderate. Thus, all 26 articles were eligible for review (Fig. 1).

### 2.4 Data abstraction and analysis

Data abstraction is collecting relevant data based on the research questions and placing them in a table. The review abstracted data from the 26 articles and performed thematic analysis by thoroughly reading the abstract, results, and discussion. The thematic analysis identifies themes and sub-themes based on counting, clustering, identifying patterns, similarities, and relationships in the abstracted data [44]. We performed three steps in generating the themes and sub-themes. The initial phase involved extracting patterns from the reviewed publications identifying seven main themes. After further analysis, these broad themes were divided, resulting in 30 sub-themes. Each theme is assigned a title that encapsulates its essence. To ensure the validity of the themes, they were developed collaboratively among the research team. Differences in opinion and conceptual difficulties were resolved through discussion until agreement was achieved on the classification of themes. Finally, after a rigorous review to ensure accuracy and relevance, we identified five main themes accompanied by 24 sub-themes. The thematic analyses have been presented, discussed, and consulted for external validation at an international conference. Their unanimous endorsement confirmed the relevance and appropriateness of the identified themes and sub-themes to the study's objectives.

Building on Shaikh et al. [45], our thematic analysis synthesises the UTAUT2 framework, representing the forefront of technology adoption theories in the Fintech sector. UTAUT2 stands as an integrative model that encapsulates previous seminal technology adoption theories—Innovation Diffusion Theory (IDT), Theory of Reasoned Action (TRA), Theory of Planned Behaviour (TPB), and Technology Acceptance Model (TAM), as outlined by Venkatesh et al. [46]. It advances the original UTAUT by incorporating three additional dimensions: habit, hedonic motivation, and price value, offering a comprehensive framework on the determinants of technology adoption behaviour. To account for Fintech adoption barriers, six consumer-perceived risk dimensions were identified, namely security, performance, financial, time, social, and psychological risk, as suggested in Ref. [47], and privacy risk as another dimension to acknowledge the increasing threats of data breach these days [[21], [25], [48], [49], [50], [51], [52]].

Concerning the crucial role of trust in the existing Fintech literature, the analysis has identified trust dimensions based on Mao et al. [53], namely cognition-based, personality-based, institutional-based, calculative-based, and experience-based trust. A quality theme was guided by the IS Success Model or the DeLone and McLean Information System Success Model, categorising quality into information, system, and service quality [54]. The fifth theme, i.e., “other”, encompasses variables that do not fall under any predefined group: attitude and experience. To assign variables to the correct sub-themes, we identified those with shared characteristics through a meticulous review and synthesis of their operational definitions and the theories underpinning them.

### 2.5 Weight analysis

Weight analysis examines the strength of a predictor (independent variable) on the outcome (dependent variable) [31]. Jeyaraj et al. [55] highlight the need for weight analysis as it allows the predictive power of an independent variable to be investigated. It was performed by dividing the number of significant relationships by the total number of analysed relationships between an independent and dependent variable [56]. Predictors can be classified into well-utilised (variables examined ≥5 times) or experimental (variables examined <5 times). If a well-utilised weight equals or exceeds 0.8, it is considered the best predictor. If not, it will be the least effective predictor. An experimental predictor is considered promising when its weight is equal to 1.

## 3 Results

### 3.1 Background and keywords

The review presents seven keywords related to Fintech banking (Table 4). Two papers conducted an electronic banking study on hospital staff and traditional counter-services users [57,50]. Only Salciuviene et al. [51] studied online financial services. There are nine studies focused on online banking in Asia [58,59,60,61,52], North America [62], Africa [63,64], and Europe [65]. Ten studies examined mobile banking in Asia [66,48,67,21,27,68,69,70], Africa [71,49], and Europe [21]. Interestingly, only Merhi et al. [21] compared vital factors that may hinder or facilitate the acceptance of mobile banking services between the two countries. Two smartphone banking studies were in Asia [72,26]. Furthermore, Saif et al. [11] is the only one to study digital-only banks. Next, only Asadi et al. [25] examined cloud computing in Malaysian banking. Against this background, the seven Fintech-related keywords hereafter refer to Fintech service in banking.

**Table 4:** Keywords in Fintech/banking methods research.

Fintech/Banking Method	Definition	Country	Studies
Electronic banking	Banking transactions without visiting a brick-and-mortar institution [57].	Jordan	[57,50]
Online financial services	Services that replaced offline branch banking through computers and mobile phones [51].	Lithuania	[51]
Online/Internet Banking	Automated banking products and services are distributed to customers directly via electronic communication [73].	Democratic Republic of the Congo	[64]
		India	[59,60]
		Taiwan	[61]
		Canada	[62]
		Indonesia	[52]
		Tunisia	[63]
		Vietnam	[58]
		Scotland	[65]
Mobile banking	A service delivery channel that allows customers to make banking transactions using mobile devices [74].	Mongolia	[67]
		Bangladesh	[66]
		Lebanon	[21]^a^
		England	[21]^b^
		Ethiopia	[71]
		Vietnam	[70]
		Philippines	[48]
		Malaysia	[69]
		Egypt	[49]
		India	[27,68]
Smartphone banking	Contains the uniqueness of computer and mobile phone features [26].	Malaysia	[72]
		South Korea	[26]
Digital-only bank	Bank that only operates online [11].	Malaysia	[11]
Cloud computing	A parallel and distributed computing system comprises interconnected and virtualised computers [25].	Malaysia	[25]

Note: 1. ^a^ Lebanon data.

2. ^b^ England data.

### 3.2 Theories and models

Fintech-banking studies employ several theories in determining consumers’ behavioural intentions (Table 5). The Technology Acceptance Model (TAM) is the most applied theory and is extended in some studies. Some combined multiple theories; for example, Asadi et al. [25] used TAM and Diffusion Theory Model (DTM) as a foundation. Meanwhile, Singh and Srivastava [27] combined TAM, Social Cognitive Theory (SCT), and the Unified Theory of Acceptance and Use of Technology (UTAUT) in their study. Recent studies used UTAUT and its extended form (UTAUT2 - hedonic motivation (HM), price value (PV), and habit) in examining behavioural intention. For example, Kaur & Arora [60] applied the UTAUT2 constructs as the mediator between perceived risk and intention. In contrast, five studies did not apply a specific model but instead made their theoretical framework comprise several factors and relationships with explicit assumptions and limitations.

**Table 5:** Theories and models.

Theory	Frequency	Studies
TAM	5	[57,58,72,26,62,64,69]
TAM-UTAUT	1	[71]
TAM-DTM	1	[25]
TAM & service dominant (S-D) logic	1	[49]
TAM-TPB (theory of planned behaviour)	1	[63]
TRA-TPB	1	[48]
TAM-SRT-UTAUT	1	[27]
UTAUT & DeLone and Mclean's ISS (Information systems success) Model	1	[68]
UTAUT	2	[59,67]
UTAUT2	2	[60,21]
Security-Risk-Trust (SRT) model	1	[61]
Mental Accounting Theory (MAT), Network Externalities Theory (NE), & Commitment-Trust Theory (CTT)	1	[11]

### 3.3 Data collection, sampling and analyses techniques

Most studies employed surveys (21), while five adopted mixed methods. Four studies utilised surveys and interviews, and one combined focus groups with interviews. Regarding sampling technique, most studies used convenience sampling [57,48,58,11,51,27]. Previous studies have applied several analysis techniques such as Structural Equation Modeling (SEM) [50,64,27], Confirmatory Factor Analysis (CFA) [57,59,65], and Partial least squares-structural equation modeling (PLS-SEM) [25,72,62]. Most studies employed CFA and SEM analysis (Table 6). Some studies used a combination of techniques to analyse the primary data. For instance, Singu and Chakraborty [68] used CFA and mediation Model 4 in their study.

**Table 6:** Data collection, sampling and analyses techniques.

Technique	Frequency	Studies
**Data Collection**		
Surveys	25	[[11], [21], [25], [26], [27], [48], [49], [50], [51], [52], [57], [58], [59], [60], [61], [62], [63], [64], [66], [67], [68], [69], [70], [71], [72]]
Interviews	5	[57,48,61,69,65]
Focus group	1	[65]
**Sampling Technique**		
Convenience	18	[57,48,58,71,67,72,60,26,62,21,49,63,51,27,68,52,70,65]
Purposive	6	[25,48,72,61,52,65]
Quota	3	[58,61,51]
Random	5	[66,59,50,64,11]
Stratified	1	[69]
**Data Analysis**		
Covariance-based structural equation modeling (CBSEM)	13	[57,71,59,60,21,63,50,64,11,27,69,70,65]
PLS-SEM	5	[25,72,62,49,52]
Regression analysis	5	[66,48,58,71,51]
CFA	13	[57,71,59,67,60,21,63,50,11,27,68,69,65]
Exploratory factor analysis (EFA)	8	[58,71,26,27,68,52,69,70]
Analysis of variance (ANOVA)	1	[51]
Correlation analysis	6	[48,58,71,61,51,69,70]
Path analysis	1	[26]
*t*-test	1	[26]
Model 4 of Hayes (2013)	2	[48,68]

### 3.4 Weight analysis

Table 7 presents variables used in previous literature in explaining the intention to use Fintech in banking. The findings show that well-utilised predictors are trust (22), PU (14), perceived security (14), PEOU (13), attitude (8), SI (7), EE (7), perceived privacy (7), and PE (6). The best predictors are attitude, trust, PU, perceived security, and PE, while the rest are the least effective. Furthermore, there are 24 promising and 11 experimental predictors.

**Table 7:** Weight analysis.

Independent Variable	Significant results	Insignificant results	Weight
Category: Best predictor (5)			
Attitude	P: [12,37,63,69,71,88^cd^] N: [71]		1
Trust	P: [12,19,20,32,46,48,51,67^ab^,69,76,83,89,90,100,103,108,111] N: [69]	[66,59,27]	0.9
Perceived security	P: [66,48,72,49,61,50,51,27,52,69] N: [21]^ab^	[59,68]	0.9
Perceived usefulness (PU)	P: [1,3,12,20,46,51,63,69,71,83,88^cd^,90]	[71]	0.9
Performance expectancy (PE)	P: [37,39,48,67^a^,100]	[21]^b^	0.8
**Category: Least Effective Predictor (4)**			
Perceived ease of use (PEOU)	P: [25,72,49,63,50,51,27,52] N: [71]	[57,66,58,26]	0.7
Perceived privacy	P: [19,67^ab^,69,83]	[66,52]	0.7
Effort expectancy (EE)	P: [37,39,67^b^,100]	[37,48,67^a^]	0.6
Social influence (SI)	P: [71,67,60]	[37,67^ab^,99]	0.4
**Category: Promising Predictor (24)**			
Perceived risk/Overall Risk (OR)	N: [60,61,70,65]		1
Habit	P: [37,67^ab^]		1
Perceived cost	P: [25,48]		1
Perceived website usability (PWU)	P: [59,52]		1
Perceived Enjoyment	P: [57,49]		1
Subjective norm	P: [57,63]		1
System quality (SYQ)	P: [68]		1
Interface design (ID)	P: [69]		1
Infrastructure quality	P: [48]		1
Service quality (SEQ)	P: [68]		1
Number of services	P: [11]		1
Information quality (IFQ)	P: [68]		1
Experience	P: [59]		1
Economic efficiency	P: [11]		1
Relative benefits	P: [52]		1
Environmental concern (EC)	P: [11]		1
Confidentiality	P: [51]		1
Perceived behavioural control (PBC)	P: [63]		1
Computer self-efficacy	P: [27]		1
Technology competency	P: [66]		1
Image	P: [68]		1
Company reputation	P: [52]		1
Perceived value	P: [11]		1
Perceived financial cost	P: [27]		1
**Category: Experimental Predictor (11)**			
Price value (PV)	P: [48,67^b^]	[21]^a^	0.7
Government support	P: [58,71]	[52]	0.7
Perceived web security	P: [64]^d^	[64]^c^	0.5
Security risk	N: [26]	[11]	0.5
Facilitating conditions (FC)	P: [67,68]	[59,60]	0.5
Hedonic motivation (HM)	P: [59,60]	[21]^ab^	0.5
Convenience	P: [11]	[50]	0.5
Value-added		[69]	0
Promotion		[69]	0
Critical mass		[11]	0
Functional Risk		[11]	0

Note: 1. The weight analysis focuses on behavioural intention studies only for brevity.

2. Promising and experimental predictors are the least examined predictors.

3. ^a^ Lebanon data.

4. ^b^ England data.

5. ^c^ Findings for online banking users.

6. ^d^ Findings for non-online banking users.

7. P means positively influenced.

8. N means negatively influenced.

9. All variables in this table are directly sourced from the original author's work. Weight analysis combines the variables that have similar meanings in alignment with the theme used in thematic analysis.

All the significant independent variables have a positive relationship with intention except perceived risk and security risk because perceiving risk as a threat deters people from using Fintech [60]. However, some researchers find the opposite conclusion to the average for trust, perceived security, attitude, and PEOU.

Table 8 highlights 34 promising mediating factors and moderating variables used in the intention model. Past studies have used PE, attitude, EE, trust, PER, SI, HM, and PV as mediating variables, while trust and perceived risk are moderating variables.

**Table 8:** Weight analysis for mediating and moderating variables.

Ind. Variable	Mediating Variable	Significant results	Weight
PEOU (EE)	PU (PE)	[26,63]	1
Enjoyment (HM)		[57]	1
Experiences		[57]	1
Perceived risk		[60]	1
Perceived security		[57]	1
Trust		[57,26]	1
Subjective norm (SI)		[57]	1
PU (PE)	Attitude	[49]	1
PEOU (EE)		[49,63]	1
Enjoyment (HM)		[49]	1
Perceived security		[49]	1
Perceived privacy		[49]	1
Enjoyment (HM)	PEOU (EE)	[57]	1
Experiences		[57]	1
Peceived security		[57]	1
Trust		[57]	1
Perceived security	Trust	[25,48,61,52]	1
Perceived risk		[48,70]	1
Perceived privacy		[25,48,52]	1
Relative benefits (PE)		[52]	1
Company reputation (SI)		[52]	1
Usability		[52]	1
Infrastructure quality (SYQ)		[48]	1
Perceived costs (PV)		[48]	1
Government support (FC)		[52]	1
Perceived security	Perceived risk (PER)	[61]	1
Trust		[65]	1
Perceived risk	SI	[60]	1
Perceived risk	HM	[60]	1
Perceived risk	PV	[60]	1
**Ind. Variable**	**Moderating Variable**	**Significant results**	**Weight**
Perceived risk	Trust	[60,70]	1
Attitude		[62]	1
PU (PE)	Perceived risk	[62]	1
Attitude		[62]	1

Note: 1. The aforementioned factors are promising mediation and moderating variables.

2. Experimental variables are excluded for brevity.

3. Promising and experimental predictors are the predictors few researchers investigated, along with trust, security, and intention to adopt Fintech.

4. All variables in this table are directly sourced from the original author's work. Weight analysis combine the variables that have similar meanings, in alignment with the theme used in thematic analysis.

### 3.5 Thematic analysis

The thematic analysis provides critical analyses by grouping the equivalent variables into similar sub-themes. Table 9 provides themes and sub-themes of the behavioural intention model. It produces five main themes, namely UTAUT2 variables, Risk, Trust, Quality and Other. The theme further produces 24 sub-themes. It shows that PSR (24), IBT (21), PE (21), EE (20), and PPR (14) are the most frequently used factors.

**Table 9:** The themes and sub-theme.

Authors	UTAUT2 variables							Risk								Trust					Quality			Other	
	PE	EE	SI	FC	HB	HM	PV	PR	PSR	PPR	SR	TR	FR	PYR	OR	CGT	PBT	IBT	CLT	EBT	SYQ	S E Q	IFQ	A T T	E P
Safari et al., 2022 [64]	/	/							/	/			/					/		/				/	
Ivanova & Kim, 2022 [67]	/	/	/	/				/	/									/							
Inder et al., 2022 [59]	/	/	/	/	/	/			/									/			/	/	/	/	/
Saif et al., 2022 [11]	/	/	/				/	/	/							/		/				/			
Singu & Chakraborty, 2022 [68]	/	/	/	/					/									/			/	/	/		
Kaur & Arora, 2021 [60]	/	/	/	/		/	/	/	/		/	/	/	/	/			/							
Van et al., 2020 [70]								/	/	/	/	/	/		/			/							
Akhter et al., 2020 [66]	/	/		/					/	/								/							
Rawwash et al., 2020 [50]	/	/							/	/								/		/		/			
Karim et al., 2020 [72]	/	/							/							/									
Mostafa, 2020 [49]	/	/				/			/	/								/						/	
Hagos & Singh, 2019 [71]	/	/	/	/														/						/	
Merhi et al., 2019 [21]	/	/	/		/	/	/		/	/						/									
Singh & Srivastava, 2018 [27]		/	/	/			/		/									/							
Asadi et al., 2017 [25]	/	/	/				/		/	/							/							/	
Chiu et al., 2017 [48]							/		/	/						/			/		/				
Ong & Lin, 2015 [61]								/	/	/			/	/	/			/	/						
Mangin et al., 2014 [62]	/	/							/	/						/		/						/	
Salciuviene et al., 2014 [51]	/	/							/	/								/							
Vaithilingam et al., 2013 [69]	/								/								/	/			/				
Susanto et al., 2013 [52]	/		/	/					/	/						/		/			/		/		
Abbad, 2013 [57]	/	/	/			/			/									/							/
Nasri & Charfeddine, 2012 [63]	/	/	/	/					/	/														/	
Kim & Kang, 2012 [26]	/	/							/									/		/					
Chong et al., 2010 [58]	/	/		/														/							
Yousafzai et al., 2009 [65]									/	/			/		/	/		/							
Total	21	20	12	10	2	5	6	5	24	14	2	2	5	2	4	7	2	21	2	3	5	4	3	7	2

PE = Performance Expectancy	PR = Performance Risk	CGT = Cognition-based Trust	ATT = Attitude	SYQ = System Quality
EE = Effort Expectancy	PSR = Perceived Security Risk	PBT = Personality-based Trust	EP = Experience	SEQ = Service Quality
SI = Social Influence	PPR = Perceived Privacy Risk	IBT = Institution-based Trust		IFQ = Information Quality
FC = Facilitating Conditions	SR = Social Risk	CLT = Calculative-based Trust		
HB = Habit	TR = Time Risk	EBT = Experience-based Trust		
HM = Hedonic Motivation	FR = Financial Risk			
PV = Price Value	PYR = Psychological Risk			
	OR = Overall Risk			

#### 3.5.1 UTAUT2 variables

UTAUT2 variables include PE, EE, SI, FC, HB, HM, and PV (Table 10) [46]. Given the similarities among variables, this study grouped them under corresponding sub-themes (Table 10). The review suggests PE significantly influences Fintech intention because it enhances job performance through improved income, operational efficiency, productivity, and flexibility [67,49,68]. However, PE is deemed insignificant by some customers [[21], [69], [71]]. EE is generally considered significant, with customers viewing Fintech as user-friendly and straightforward. Nonetheless, a minority of studies [[21], [58], [59], [60]] report EE as having an insignificant impact. The influence of SI is pronounced among customers without prior experience, who are heavily swayed by their social circles, including friends, mass media, experts, and other influential figures [71,67,60,63]. However, for some users, SI holds little significance [59,21,11,27]. Additionally, the propensity to use Fintech is heightened when it offers built-in facilities that integrate seamlessly with existing applications and technologies [66,63]. However, a few studies found the FC effect negligible [59,60,52]. Merhi et al. [21] discovered that engaging mobile banking features can lead to habitual use. HM is also a driving force, with consumers drawn to the fun, enjoyment, and excitement Fintech provides [59,60,49], but an insignificant of HM is observed in Merhi et al. [21]. Lastly, PV is influential when customers perceive the benefits to outweigh the monetary costs, making Fintech reasonably priced [[11], [21], [60]]. However, when there are additional hardware charges, the impact of PV is deemed insignificant [21].

**Table 10:** Scope of UTAUT2 variables.

Scope	Operational Definition	Frequency
Performance Expectancy (PE) [including Perceived usefulness (PU), Relative benefits (RB), Value-added, Economic efficiency, Promotion (PM), and Environmental concern (EC)]	• PE (UTAUT): How technology improves job performance [68]. • PU (TAM): The extent to which a particular system would enhance a consumer's ability to perform effectively [72]. • RB: Perception of new services benefits more than existing ones [52]. • Value-added: The degree to which mobile banking contributes value to consumer experience [69]. • Economic efficiency: How the technology can minimise the expenditure of time, energy, and money [11]. • PM: An essential marketing technique to communicate the advantages of using mobile banking to customers [69]. • EC: The understanding and consciousness of environmental matters [11].	21
Effort Expectancy (EE) [including Perceived ease of use (PEOU) and Convenience]	• EE (UTAUT): Degree of ease while using the system [60]. • PEOU (TAM): The degree to which the customer perceives a system is user-friendly and straightforward to learn and use [27]. • Convenience: Always have access to the services anywhere [50].	20
Social Influence (SI) [including Subjective norm (SN), Critical mass, Image, and Company reputation (CR)]	• SI (UTAUT): The influence of essential people on one's decision [21]. • SN (TRA): Perception of whether the people who are significant to them believe they should or should not engage in a specific behaviour [57]. • Critical mass: The belief that the quantity of individuals using the technology is substantial enough [11]. • Image (IDT): A belief that adopting technology can improve one's image and social status [68]. • CR: The perception that a company is both widely recognised and held in high esteem [52]	12
Facilitating Conditions (FC) [including Perceived behavioural control (PBC), Computer Self-efficacy, Technology competency, and Government support (GS)]	• FC (UTAUT): Technical and organisational infrastructure is available to support the system [68]. • PBC (TPB): The extent to which consumers believe they can control and influence their actions [63]. • Computer self-efficacy (SCT): Individual's belief to use technology [27]. • Technology competency: The level of knowledge and skill a person possesses in using technology [66]. • GS: Government's assurance regarding system security and technological infrastructure [71].	10
Habit (HB)	HB (UTAUT2): Unconscious action from learned behaviour through repeated use of technology [21].	2
Hedonic Motivation (HM) (including Enjoyment)	• HM (UTAUT2): Enjoyment and pleasure [46]. • Enjoyment: The satisfaction of using a computer [57].	5
Price Value (PV) [including Perceived value, Perceived cost (PC), and Perceived financial cost]	• PV (UTAUT2): A tradeoff between the perceived benefit and cost of using IT [21]. • Perceived value: Assessing the benefits and costs offered [11]. • PC: The extent to which individuals perceive Fintech service as costly [48]. • Perceived financial cost: The expenses associated with mobile banking activities [27].	6

Note: 1. UTAUT is the Unified Theory of Acceptance and Use of Technology.

2. TAM is the Technology Acceptance Model.

3. TRA is the Theory of Reasoned Action.

4. IDT is Innovation Diffusion Theory.

5. TPB is the Theory of Planned Behaviour.

6. SCT is the Social Cognitive Theory.

#### 3.5.2 Risk

Risk is believing in negative consequence probabilities to pursue the desired outcome [70]. Perceived risk consists of PR, PSR, PPR, SR, TR, FR, and PYR (Table 11). Saif et al. [11] used functional risk to represent PR. Customers will avoid Fintech in banking, which has low download speed, technical problems, and unscheduled website maintenance [60,61,70]. Only Saif et al. [11] found PR to be insignificant. System security is the most concerning aspect in influencing customers [66,49,61,27] and non-users [64]. However, several researchers [59,64,11,68] found that system security is insignificant for some users. Privacy issues have gained prominent concern due to online data storage, which is subject to potential data breaches and theft. Customers prefer Fintech if their privacy is highly protected [48,21,51].

**Table 11:** Scope of risk variables.

Scope	Operational Definition	Frequency
Performance Risk (PR) [including functional risk]	• PR: Factors distorting the efficiency of the Internet banking system [60]. • Functional risk: The probability of customers experiencing issues due to the instability of the systems [11].	5
Perceived Security Risk (PSR) [including perceived security and perceived web security]	• PSR: Loopholes in the security system result in unauthorised access to customer data [70]. • Perceived security: The risk associated with various incidents, such as unauthorised access [72]. • Perceived web security: A comprehensive approach to safeguarding online activities and operations [64].	24
Perceived Privacy Risk (PPR) [including perceived privacy and Confidentiality]	• PPR: The customer's personal information is used without consent [70]. • Perceived privacy: The perspective on the collection of sensitive information by businesses and the illicit utilisation of the data they have gathered [66]. • Confidentiality: The protection of sensitive information [51].	14
Social Risk (SR)	SR: Potential social status loss due to adopting products or services [60].	2
Time Risk (TR)	TR: Time-consuming when using the systems [60].	2
Financial Risk (FR)	FR: Financial losses from information and transaction errors [70].	5
Psychological Risk (PYR)	PYR: Possible loss of self-esteem [60].	2

Past researchers found SR to be a significant variable [60,70]. Consumers use Fintech to get acceptance, recognition, and a sense of belonging with their social circle (friends, family, and colleagues). Researchers [60,70] agreed that TR is a significant factor. Users refuse Fintech as it is time-consuming and requires knowledge and self-efficacy. They also believe there is a low tendency to recover losses in high-risk transactions [60,61,70,65]. Customers believe the Fintech experience will develop anxiety and tension, especially during technical errors and cybercriminal incidents [60,61]. The discomfort will reduce their trust, thus discouraging them from adopting it.

#### 3.5.3 Trust

Trust measures customers' confidence in banks to provide adequate services competently and with integrity [49]. Trust can be categorised into CGT, PBT, IBT, CLT, and EBT (Table 12) [53]. IBT is the most influential factor affecting customers' intention to use Fintech in banking [57,58,26,61,65]. A bank must be dependable, capable, secure, and transparent in acquiring customers’ trust, especially in a virtual environment. However, trust is insignificant for some [66,27]. In addition, trust only moderates attitude-intention but not the usefulness-intention relationship [62].

**Table 12:** Scope of trust variables.

Scope	Operational Definition	Frequency
Cognition-based Trust (CGT)	CGT: Feelings and rational assessment [75].	7
Personality-based Trust (PBT)	PBT: Established from individual personality psychology [76].	2
Institution-based Trust (IBT)	IBT: The belief that the technology provider has appropriate structures and standards [77].	21
Calculative-based Trust (CLT)	CLT: Cost-benefits of economic value evaluation [78].	2
Experience-based Trust (EBT)	EBT: Experience and social-exchange knowledge with another party [76].	3

#### 3.5.4 Quality

Quality is categorised into SYQ, SEQ, and IFQ, as outlined in Table 13. Two studies [59,52] use usability to represent quality. Interface design and infrastructure quality represent SYQ. An appealing Fintech system includes accessible, well-designed, secure, error-free, and high-speed [68,69]. SEQ is favourable, particularly for its convenience and breadth of services, as it meets user expectations and heightens satisfaction [11,68], though this is only sometimes agreed upon [50]. IFQ is deemed essential due to the accuracy, timeliness, relevance, usefulness, and completeness of the information provided [68], which is essential for banks to maintain their clientele.

**Table 13:** Scope of quality variables.

Scope	Operational Definition	Frequency
System quality (SYQ) [including Interface design (ID) and Infrastructure quality (ISQ)]	• SYQ: The extent to which the system can easily support users' transactions [68]. • ID: The capacity to receive messages with improved attributes [69]. • ISQ: Connection speed and mobile signal [48].	5
Service quality (SEQ) [including Number of services]	• SEQ: Service delivery regarding time precision, prompt responses, and professional and personalised services [68]. • Number of services: The perception that technology provides diverse essential and additional financial services [11].	4
Information quality (IFQ)	IFQ: Measures website information quality [68].	3

#### 3.5.5 Other

There are two “other” factors: ATT and EP (Table 14). Consumers have a positive attitude toward Fintech as it makes sense and is easy and cost-effective [62,63,64]. However, one research found that attitude negatively influenced intention [71]. In addition, consumers intend to use electronic banking when they have developed experience using the systems [59].

**Table 14:** Scope of individual factors variables.

Scope	Operational Definition	Frequency
Attitude (ATT)	ATT (TRA): Positive or negative thoughts toward specific behaviour [25].	7
Experience (EP)	EP: A learning process through experience acquiring the necessary skills to use the new system [57].	2

Note: 1. TRA is the Theory of Reasoned Action.

### 3.6 Overall framework

**Fig. 2:**
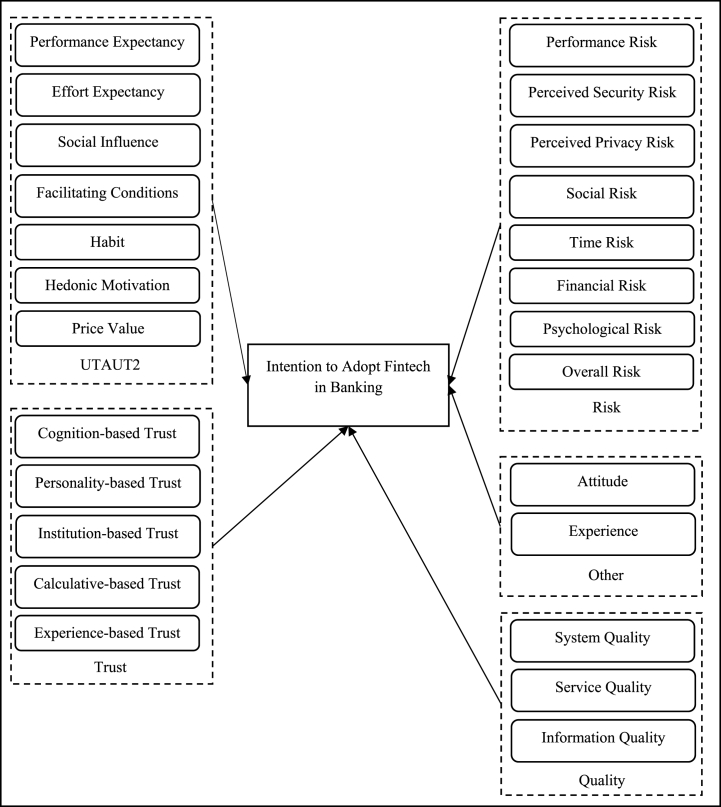
Direct effect framework.

**Fig. 3:**
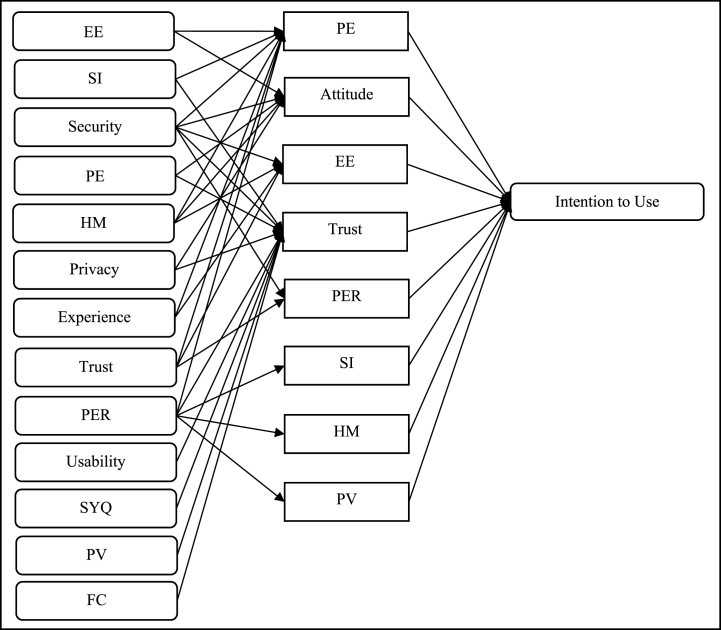
Mediating effect framework.

**Fig. 4:**
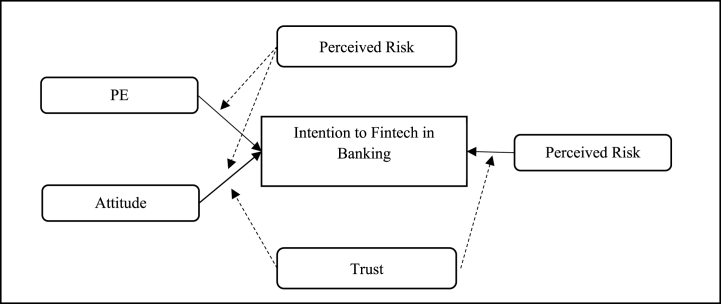
Moderating effect framework.

The review identifies 24 determinants influencing intentions towards Fintech in banking. Within these, trust emerges as a multifaceted construct encompassing five distinct elements. Security, intriguingly, is positioned as a subset of perceived risk. The influence of trust and security on Fintech adoption intentions is nuanced, manifesting directly and indirectly. Fig. 2 illustrates the direct effect of Fintech intention determinants, while Fig. 4 presents the indirect (moderating) role of trust and perceived risk. Fig. 3 shows that the relationship between EE, PE, SI, HM, experience, security, privacy, PER, trust, usability, SYQ, PV, FC, and intention also have been mediated by several factors (PE, Attitude, EE, Trust, PER, SI, HM, PV). Fig. 4 shows that the relationship between PE, attitude, perceived risk, and intention has also been moderated by several factors (Perceived risk, Trust).

#### 3.6.1 Direct effect

Refer Fig. 2.

#### 3.6.2 Mediating effect

Refer Fig. 3.

#### 3.6.3 Moderating effect

Refer Fig. 4.

## 4 Discussion

The best well-utilised predictors are PE, trust, perceived security, attitude, and PU. Among the UTAUT2 variables, PE/PU are strong predictors in influencing customers’ behavioural intention. Customers believe service providers are unwilling to deliver valuable services if they do not achieve performance expectations [79]. Moreover, customers prefer beneficial technology that enhances their banking experiences [80].

Institutional trust is the most influential category due to lack of personal contact, anonymity, and security issues [76,81,82]. It encompasses the subjective belief that institutions, particularly banks in this case, will act in ways consistent with positive expectations, fostering confidence in their operations and security measures. It also covers website information quality, consumers’ privacy protection and security, and operational stability of institutions, which new institutions lack [83]. For example, customers will lose trust in banks with low-security features [47]. Institutional Trust Theory (ITT) is a theoretical framework that underpins this concept. ITT is essentially the belief that individuals hold in the reliability and integrity of the institutions they interact with, and it is considered a fundamental condition for customers to engage with electronic platforms and services. The importance of ITT is highlighted by research that has shown its significant influence on customers' continuance intention, with studies like Ofori et al. [84] demonstrating that ITT can account for a substantial portion of the variance in users' intentions to continue using internet banking. Customers are also wary of banks exploiting their vulnerabilities [85]. Trust in adopting information technology and electronic-based services cannot be underrated [84]. Thus, service providers must exercise caution when building encrypted services that include multiple security checks and effective fraud detection techniques to secure customer trust [86].

Perceived security and privacy are the most critical risks in online transactions due to data thefts from cybercriminal threats such as malware and phishing attacks [87,88]. Data breaches cause economic losses to users and financial institutions, leading to a loss of trust in the banking sector [89]. When customer data is compromised, it can lead to financial fraud, identity theft, and other malicious activities. Such incidents erode trust in the banking sector, as customers may question the ability of financial institutions to protect their information. Lack of trust and unhappiness are the primary reasons clients switch financial institutions [90]. Thus, research, awareness, education, and training programmes are essential to protect customers' financial security against cyberattacks [91]. These initiatives aim to empower consumers and banking professionals with the knowledge and skills to recognise and mitigate cyber risks. Informed consumers will pay more attention to potential cyber threats, thus lowering the incidence of cybercrime [92]. In addition, financial institutions need to invest in robust security systems to safeguard their customers and operations. Enhanced security system such as firewalls, authentication and password, multilayer inspection gateways, and extra customer service is vital for protecting customers [60]. These technologies work together to defend against cyberattacks and unauthorised access.

Attitude is a crucial individual factor influencing Fintechs’ intention, particularly in the IT industry [93]. A positive attitude can be developed when individuals perceive technical breakthroughs as applicable [94]. As customers learn the essential Fintech skills, their judgment of the system will improve, thus motivating usage. However, the positive influence of attitude towards intention to use Fintech depends on PU, PEOU, enjoyment, and perceived security and privacy [49]. For example, the PU of technology in efficiency, productivity, functionality, and flexibility will enhance job performance, thus inculcating a positive attitude. Besides, customers will develop a positive attitude if they believe technology is easy and less complex, which refers to PEOU.

## 5 Recommendation and future direction

The 26 articles reviewed various theories, theoretical models, and constructs. It also provides a comprehensive picture of all factors explored in behavioural intention research from 2009 to 2022 and lays the framework for future research. Following the criteria of past studies, the Theory, Context, Constructs, and Methodology (TCCM) framework is utilised to give recommendations for future research [95,33], as illustrated in Table 15.

**Table 15:** Future research agenda.

TCCM	Future research questions	Reference
Theory (5)	Future research may expand on the latest model, UTAUT2.	A
	Underutilised theories should be considered in future studies.	A
	Future research should integrate two or more theoretical frameworks.	[96,97,98,99,100]
	Future studies may explore the indirect role of trust and security in shaping the link between Fintech antecedents and user intentions.	A
	Future studies should introduce the complex interactions of moderating and mediating effects.	A
Context (6)	Future research should delve deeper into relatively unexplored domains, such as digital-only banking.	A
	Future research should focus on rural populations.	A
	Future studies may delve into nuanced barriers consumers face.	A
	Future research must broaden its scope to encompass the emerging socio-economic challenges.	A
	Future research may explore the psychological profiles of consumers.	A
	Future studies may focus on new generations' needs, expectations, and preferences.	[48]
Construct (2)	Future research should take a holistic approach to examining risk, trust, and quality roles in Fintech adoption.	A
	Future research should focus on promising factors corresponding to the growing issue of meeting consumer expectations.	A
Method (3)	Future research should explore mixed methods.	A
	Future research should consider employing a triangulation method.	[24]
	Future studies may collect data using a random sample technique.	[60,49]
	Future studies may employ big data analysis.	A
	Future research should focus on utilising longitudinal methods.	A
	Future research may use comparative empirical evidence like MGA.	A
	Future studies should consider hybrid review and meta-analysis.	A

Note: 1. A means authors suggestion.

### 5.1 Theory

The review discovered that TAM is the most applied theory while increasing studies use UTAUT and their modifications to explain the role of trust, security, and determinants of Fintech behavioural intention among banking consumers. Despite the usefulness of TAM and UTAUT, there is room for improvement in encapsulating consumer-related variables comprehensively [101]. Recently, research has gravitated towards the UTAUT2 model to elucidate customers’ technology acceptance behaviour [102,103]. Focusing on the Technology adoption model aligns with Mullan et al. [104], suggesting that broad-spectrum theories and models (such as the Theory of Reasoned Action and the Theory of Planned Behaviour) may be less insightful than those tailored explicitly to Fintech innovation. Past literature has expanded TAM and UTAUT theories by integrating new elements; however, significant opportunities for theory development remain. In the evolving landscape of Fintech (such as the COVID-19 pandemic, cashless society, artificial intelligence, and machine learning), it is imperative to attain a holistic grasp of the determinants influencing its adoption, particularly by accommodating the distinct dynamics inherent to Fintech innovations and reflecting diverse stakeholder viewpoints. Consequently, updated research models are crucial, ensuring relevance and addressing current and future challenges in Fintech adoption.

While the expansion of theoretical frameworks in understanding trust and security effect on Fintech use is notable, a more profound insight requires the synthesis of diverse theoretical perspectives, as suggested by literature [88,49,61,11,27]. Integrating underexplored theories like Service-Dominant Logic, ISS DeLone and McLean, Trust-Security Risk Model, Mental Accounting Theory, Network Externality Theory, Commitment Trust Theory, Institutional Trust Theory (ITT) [76] and Social Cognitive Theory (SCT) [105] with established technology adoption models could yield a more nuanced understanding. Future research should integrate two or more theoretical frameworks to develop an extensive mode [96,97,98,99,100]. The technology adoption model in isolation can only provide a limited perspective. Such an approach would transcend the limitations of a single-theory model, providing a multi-dimensional view of user behaviour that captures the complexity of technology adoption in practice. Future research leveraging this integrative approach could produce models that are both exhaustive in scope and attuned to the intricacies of real-world application.

The existing Fintech adoption model lacks empirically validated methods that account for moderating and mediating effects. Notably, there needs to be more research on the indirect role of trust and security in shaping the link between Fintech antecedents and user intentions. Integrating such effects offers fertile ground for academic inquiry [60,62,70]. Recent studies indicate that further exploration is warranted into the mediating effects of Performance Expectancy (PE), Effort Expectancy (EE), Social Influence (SI), Hedonic Motivation (HM), Price Value (PV), attitude, trust, and perceived risk [57,25,60,26,49,63,65], as well as the moderating effect of perceived risk [62]. This approach may uncover more profound insights into the complexities of Fintech adoption. Future researchers should create models that embed these complex interactions, increasing our understanding of the nuanced dynamics of Fintech adoption and benefiting a more comprehensive range of stakeholders.

### 5.2 Context

The review found that Fintech research in developing countries has predominantly focused on mobile [66,71,21] and online banking [59,64,52]. However, areas such as digital-only banking and cloud computing still need to be more examined. This oversight is particularly notable in emerging markets characterised by low Fintech development. Further investigation is warranted to understand consumer acceptance in these contexts, where digital advancements can significantly contribute to social welfare, consumer well-being, and financial inclusivity. Critically, studies focusing on rural populations, where Fintech adoption is minimal, are essential to provide valuable insights for policymakers and financial managers, informing policy reforms and the design of services tailored to enhance Fintech infrastructure in underserved areas.

Moreover, the existing literature presents significant gaps in addressing the intricacies of user resistance and the implications of cybersecurity incidents [17]. While prior studies have investigated Fintech features and benefits (such as PE, EE, FC, and quality), there needs to be more research delving into the nuanced barriers consumers face. Notably, cognitive resistance to Fintech adoption, often exacerbated by external factors such as media influence and economic trends (i.e., BRICS agreement), requires further scholarly attention [16,106]. Moreover, the ever-evolving techniques of cybercriminals to circumvent legal barriers and breach security systems, like the Unicredit case in 2017, cast a shadow over user trust and perception of security, pivotal elements that warrant comprehensive investigation [107,18]. Therefore, future research must broaden its scope to encompass these emerging socio-economic challenges, offering insights that can inform the development of a robust regulatory and supervisory framework for Fintech. Such efforts are essential for securing consumer trust and strengthening the reputation of Fintech solutions [108].

To our knowledge, comparative research on Fintech across different countries is scant, with Merhi et al. [21] being a notable exception. Chiu et al. [48] have highlighted the value of cross-national studies for benchmarking and theory validation. Such comparative work is crucial for discerning how cultural dimensions (such as individualism/collectivism, uncertainty avoidance, masculinity/femininity, and power distance) [109], demographic factors (including gender, age, profession, and income), and levels of financial access (distinguishing between banked and unbanked customers) shape consumer perceptions of Fintech. Furthermore, exploring the psychological profiles of consumers, such as their risk preferences (risk-seeking, risk-averse, risk-neutral) and innovativeness (innovators, early adopters, early majority, late majority, laggards) can enhance our understanding of the Fintech adoption phenomenon. Besides, future research should also address the distinctive needs, expectations, and preferences of new generations, such as millennials, Generation Z, and Generation Alpha, to yield fresh perspectives [110]. Consequently, investigating adoption behaviours among diverse samples is a pivotal new research agenda.

### 5.3 Construct

The thematic analysis of 26 articles reveals that the constructs most frequently used (>10 times) include perceived security risk, institution-based trust, performance expectancy, effort expectancy, perceived privacy risk, and social influence. These frequent findings reflect the predominant application of the TAM and UTAUT models in Fintech adoption research. We harmonised corresponding constructs from TAM with those of UTAUT, recognising that perceived usefulness (PU) is comparable to performance expectancy (PE), perceived ease of use (PEOU) correlates with effort expectancy (EE), and subjective norm is consistent with social influence (SI). This consolidation enhances clarity and consistently identifies Fintech adoption determinants, demonstrating UTAUT's compatibility in assimilating earlier technology adoption theories.

Moreover, the review indicates that perceived security is pivotal in influencing Fintech adoption, but inadequate security measures can amplify perceived risks and deter adoption. The impediments to Fintech adoption arise from security risk and a spectrum of risk dimensions such as performance, security, privacy, social, time, financial, and psychological risks [60,70], neglecting their cumulative impact on adoption intentions. Similarly, trust is multifaceted, encompassing cognitive-based, personality-based, institutional-based, calculative-based, and experience-based dimensions of trust [53]. Research, however, has disproportionately emphasised institutional trust while paying minimal attention to other trust dimensions [66,58,71,59,63]. Similarly, while quality dimensions in information systems, as outlined in models like the IS Success Model or the DeLone and McLean Model, cover system, service, and information quality [54], studies have favoured the former two [48,50,11,52,69]. Therefore, future research should take a holistic approach in examining risk, trust, and quality roles in Fintech adoption to dissect their complex interrelations and combined effects. This consideration is crucial, given that trust and perceived risk are known to modify different aspects of the adoption process: trust mediates the relationship between security and adoption intentions [25,48,61,52] and moderates the attitude and perceived risk relationship [60,62,70]. Recognising the variety in these dynamics is essential, considering the differential impact the many aspects of risk and trust have on Fintech adoption.

While the thematic analysis identified the gaps, such as which variable has been used extensively and rarely, weight analysis suggested the well-utilised, promising, and experimental predictors that could be a reference for future studies in developing a Fintech adoption determinants framework. The findings established five salient Fintech antecedents, namely attitude, PU, trust, PE, and perceived security. Meanwhile, promising predictors like reputation [52] and environmental concern [11] underscore the emerging concern regarding ethical standing and sustainability practices. As consumer values shift towards greater corporate responsibility and environmental stewardship, these elements are becoming crucial in shaping user engagement and adoption. Future research should explore these concerns to remain aligned with changing consumer expectations and sustain market relevance.

### 5.4 Method

Previous research has predominantly relied on quantitative data collection methods, primarily through surveys, while underutilising other valuable techniques such as experiments and qualitative methods like interviews and focus groups. Future research should employ mixed methods to advance the field. Roh et al. [24] recommend a triangulation approach for enhancing research quality and credibility, involving data gathering from diverse sources, including quantitative and qualitative data. This approach entails conducting semi-interviews and supplementing them with secondary data from industry reports, surveys, academic studies, social media analytics, and governmental data sets. Such a comprehensive data collection approach enriches the existing literature with enhanced reliability and validity of research findings, offering a more holistic perspective and deeper insights into the subject matter.

Most studies used convenience sampling, limiting its representativeness and generalisability of the total population. Future research should consider adopting random sampling methods, as Kaur and Arora [60] and Mostafa [49] suggested. Additionally, embracing big data analysis tools such as machine learning can be beneficial to measuring the strength of the impact of all the identified factors. Machine learning algorithms can extract valuable intuitions from exhaustive and un financial datasets such as financial transactions, user behaviour and market dynamics to uncover hidden patterns and associations [111]. For instance, it can suggest which Fintech attributes are embraced or resisted by specific user segments. This data-driven approach empowers regulators and Fintech service providers to enhance user experiences and customise services.

The review underscores that while research frequently focuses on consumer intentions toward Fintech adoption, there is a need for more empirical evidence on the consistent and long-term use of Fintech services. Studies predominantly use cross-sectional designs; thus, future research would benefit from longitudinal methods to deepen our understanding. For example, researchers could track the ongoing Fintech engagement of individuals previously studied for their initial adoption intentions. Such a longitudinal study would shed light on how Fintech usage patterns evolve and provide a richer picture of consumer behaviour within the Fintech sector, which is crucial given that the sustainability of Fintech hinges on users' continued engagement [112].

Other than that, prior research examined on single context or group, except Merhi et al. [21], who conducted a comparative analysis between England and Lebanon. Future research must provide comparative empirical evidence, such as using the Multiple Group Analysis (MGA) to examine variations in adoption factors across diverse contexts or groups. In addition to country differences, MGA provides insights concerning cross-cultural differences, age cohorts, gender preferences, income and education effects, and comparative analysis of Fintech services [113]. MGA enables tailored strategies, promoting Fintech adoption and aligning services with user preferences.

We recommend using additional methods like hybrid review and meta-analysis for scholars undertaking review studies. Hybrid review utilises systematic and narrative methods to offer a comprehensive understanding that enriches the quantitative synthesis with qualitative insights [114]. In contrast, meta-analysis consolidates from various studies to enhance the evidence base of systematic reviews, yielding precise summaries of effect sizes that lead to more reliable conclusions [56]. These versatile approaches offer valuable insights and a framework for comprehensively examining and synthesising the empirical literature in the Fintech field.

## 6 Conclusion

This study aims to provide a systematic literature review on the effect of trust, security, and other factors on consumers' intention to use Fintech in banking. The findings offer implications for the body of knowledge and management. It highlights the importance of the consumer perspective in the growth of the Fintech and banking industries. The best predictors of consumers' intentions are PE, trust, security, PU, and attitude. In addition, the findings inform researchers on specific areas and contents related to the adaptation of fintech-based innovations in banking. It highlights the research gaps and recommendations based on the TCCM framework for future studies. It also highlights promising variables influencing customers' intention to adopt Fintech in banking. The findings conclude that consumers will show interest in adopting Fintech if the system is useful, trustworthy, and secure. In addition, attitude plays a significant role in influencing consumers’ intention to use technology.

As we delve deeper into the evolving landscape of financial technology, the adoption of blockchain technology emerges as a cutting edge that will drive digital innovation [22]. Blockchain has several advantages, including immutability, anonymity, traceability, transparency, and decentralisation. However, it is crucial to note that these advantages can also be perceived as potential weaknesses, as they may raise concerns related to security and privacy. For instance, blockchain traceability and transparency might trigger privacy concerns since all blockchain network users can access the entire transaction record. The relevant parties, especially policymakers, bankers, and researchers, can plan short-term and long-term strategies to improve the acceptance of Fintech services. Fintech service providers need to emphasise the benefits of Fintech in banking and improve the system and features to match the consumers’ expectations and abilities. They must ensure that Fintech systems have security, efficiency, and effectiveness features. Policymakers have a pivotal role to play by crafting regulations supporting blockchain adoption and ensuring its responsible and ethical use. Robust regulation is warranted to secure public trust and, thus, boost the growth of Fintech and the banking industry. It will help transform society into a cashless life and promote digital banking, especially during emergencies like the recent pandemic.

## Data availability

Data are publicly available on respective databases (Scopus and WoS).

## CRediT authorship contribution statement

**Johan Ariff Jafri:** Writing - original draft, Methodology, Investigation, Formal analysis, Data curation, Conceptualization. **Syajarul Imna Mohd Amin:** Writing - review & editing, Validation, Supervision, Project administration, Funding acquisition, Conceptualization. **Aisyah Abdul Rahman:** Writing - review & editing, Validation, Supervision. **Shifa Mohd Nor:** Supervision, Software.

## Declaration of competing interest

The authors declare the following financial interests/personal relationships which may be considered as potential competing interests: Syajarul Imna Mohd Amin reports financial support was provided by Ministry of Higher Education.

**Acknowledgement**

The authors appreciate the insightful comments provided by the anonymous reviewers and acknowledge the financial support received from the Ministry of Higher Education (MoHE), Malaysia, under the Fundamental Research Grant Scheme (FRGS) [Grant code: FRGS/1/2020/SS01/UKM/02/1].
